# Composite binocular perception from dichoptic stimulus arrays with similar ensemble information

**DOI:** 10.1038/s41598-018-26679-9

**Published:** 2018-05-29

**Authors:** Oakyoon Cha, Randolph Blake, Sang Chul Chong

**Affiliations:** 1 Graduate Program in Cognitive Science, Yonsei University, Seoul, 03722 Korea; 2 Department of Psychology and Vanderbilt Vision Research Center, Vanderbilt University, Nashville, TN 37240 USA; 3 Department of Psychology, Yonsei University, Seoul, 03722 Korea

## Abstract

We view the world through laterally displaced eyes that generate two streams of image signals differing slightly in their perspectives of the visual scene. The brain derives three-dimensional structures from these two image streams by establishing binocular matches and computing image disparities between the two eyes’ views. Since the binocular matching problem can have multiple, alternative solutions, vision relies on several strategies to determine the most probable matches. The current study investigated whether the visual system might utilize regularities among neighbouring features (feature ensembles) when confronting this problem. We hypothesized that binocular perception with unlikely, anomalous ensembles would indicate unsuccessful binocular matches. We made dichoptic stimulus arrays of coloured circles and manipulated the colour similarity of stimulus items to produce probable or unusual ensembles when superimposed. Using binocular rivalry as a proxy index, we found that composite perception of dichoptic arrays was more stable when the stimulus items shared similar colours, and that unusual ensembles induced binocular rivalry. Our results suggest that binocular ensembles can be utilized to detect unsuccessful binocular matches, thus uncovering a potentially useful supplemental strategy for identifying binocular matches when viewing potentially confusing visual scenes containing redundant visual features.

## Introduction

The sensory information that culminates in our experiences of the visual world originates from the pair of retinal images formed on our two laterally displaced eyes. Ironically, however, binocular vision provides little hint of those dual monocular origins. Instead, the brain effectively melds the two views into a single, unitary experience enriched by the robust sense of stereopsis that emerges from the slight differences in perspective associated with our laterally placed eyes^1^. This seemingly automatic, effortless accomplishment of binocular integration, in fact, is the culmination of complex pattern-matching processes that (i) establish correspondences between features imaged in the left and right eyes and (ii) compute disparities associated with those features. Much work over the last several decades has focused on examining these processes and their neural concomitants (see review in^2^).

The binocular matching problem is complicated by the existence of multiple possible matches among monocular features, as famously exemplified by random-dot stereograms^3^. Theoretical work dating back to the 1970s has proposed several potential strategies for resolving these ambiguities in ways that enable a unique solution to the matching problem. One class of models exploits the so-called continuity constraint (i.e. the tendency for variations in surface depth to be gradual and smooth) to limit the acceptable range of disparity possibilities, doing so through inhibitory interactions among disparity detectors to squelch false matches^4–6^ or by favouring gradients of disparity that typify most binocular viewing situations^7^. In a related vein, other models address the matching problem through algorithms that favour disparity values defined by the object/surface boundary edges of the two monocular half-images^8^ or constraints associated with the existence of monocular occlusion boundaries^9,10^. Still other models propose that matching and disparity computation are performed on coarse and, then, fine spatial scales so that possible matches are successively delimited to smaller and smaller disparities in a manner that minimizes false matches^11–14^. Discussions of the strengths and weaknesses of those approaches have received considerable attention over the years (e.g.^15,16^).

In this paper, we explore whether binocular matching might also capitalize on regularities in the colours of a composite of features in local regions of the visual scene. The idea emerges from a simple, real-world property of textured surfaces: neighbouring surface markings comprising a surface are likely to be similar in colour owing to the inherent nature of many of the surface materials in our world. Although this characteristic is not inviolate, it does seem sufficiently general that it could well constitute a constraint to be deployed by the binocular visual system in the process of binocular matching. Our interest in this possibility was inspired, in part, by realization that humans are remarkably adept at registering ensemble properties of numerous visual items that vary along any one of a number of visual dimensions including colour^17–20^. In the following two paragraphs, we describe the colour arrays we developed to pursue this question and, then, we explain the procedure employed to assess the extent to which those arrays support successful binocular matching.

**Figure 1:**
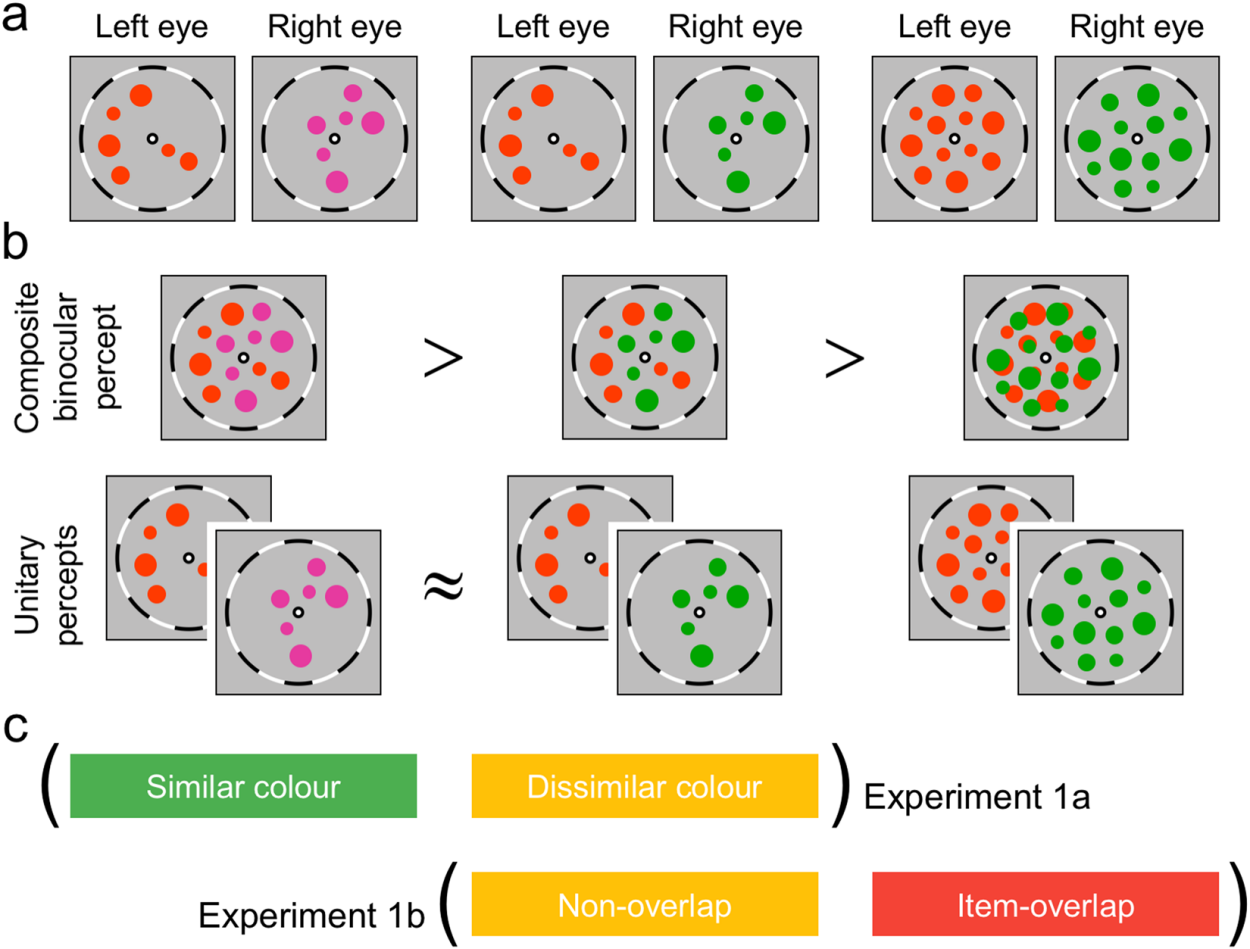
Conceptual examples of dichoptic stimulus arrays are shown in (**a**). For the presentation purpose, coloured circles are drawn in relatively bigger sizes compared to the bulls-eye fixation points and fusion frames. These dichoptic arrays produced different binocular ensembles depending on perceptual experience (composite binocular percept or unitary percept) and experimental manipulations (**b**). Superimposed images are shown as an ideal situation of composite binocular percept. Inequality and approximate equality signs in (**b**) depict our predictions on the stability of non-unitary/unitary percept. Composite binocular perception (CBP) would be more stable if it comprises similar colour circles rather than dissimilar colour circles. The latter CBP may indicate unsuccessful binocular matches. During binocular rivalry, however, unitary percepts to either left- or right-eye arrays would comprise the same colour items, and thus their stability would be similar. The colour similarity between dichoptic stimulus arrays (left and middle columns) and non-overlap vs. item overlap (middle and right columns) were manipulated and tested independently in Experiments 1a and 1b (**c**).

The stimuli we utilized were two dichoptic arrays of small, coloured circles, with the left- and right-eye arrays sometimes being highly similar in colour (e.g., all circles being reddish or all being greenish; Fig. 1a, left image pair) and other times being conspicuously different in colour (e.g., red viewed by one eye and green by the other eye; Fig. 1a, middle image pair). These displays were created following the lead of a previous study showing that stimulus arrays comprising numerous items are processed as an ensemble rather than by individual objects^21^. By using ensembles of small circles, we could insure that the two dichoptic ensembles appeared within the same region of the left- and right-eye visual fields while, at the same time, making it possible for the individual circles to fall on non-corresponding retinal areas or on corresponding retinal areas of the two eyes (conditions illustrated in Fig. 1a). To make non-overlapping arrays of circles, we made a single array of coloured circles and then selectively occluded the circles depending on the eye to which an array was presented. Half of the circles were occluded to make an array for one eye, and the other half to make an array for the other eye. Using this way, circles in the dichoptic arrays could be prohibited from being imaged in corresponding retinal areas.

How did we assess binocular matching of these arrays? Previous studies on binocular matching relied on stereo-depth judgments (e.g.,^8^) or disparity limits for binocular fusion (e.g.,^22^). In our study, we adopted a complementary approach that used binocular rivalry^23,24^ as a proxy index of *failure* to achieve binocular matches. Binocular rivalry occurs when monocular stimuli are insufficiently similar to promote stable binocular single vision – this failure is conspicuously evident by alternations in perceptual dominance between the two dissimilar stimuli. An earlier study showed that binocularly conflicting global patterns do not trigger binocular rivalry in the presence of matching local features within left- and right-eye views^25^. Here we turn that strategy around to ask whether binocular ensemble properties influence the incidence of rivalry when local features within the left and right eye views *do not* match. One might question this approach on grounds that during ordinary binocular viewing, the left- and right-eye features are usually highly similar. However, there are many real-world situations where the two eyes’ views create incompatible monocular content, including situations where the two eyes assume a convergence angle that is inappropriate for the viewing situation^26^, where one eye’s view is partly occluded^27^, and where densely textured transparent planes preclude unique matches^28^.

In the current study, we controlled local feature matches by selectively occluding stimulus items from dichoptic arrays. Thus, stimulus items in the non-overlapping dichoptic arrays were not imaged in the same/corresponding retinal areas, allowing those arrays to be superimposed to make the original single array (Fig. 1b, left and middle images in the upper row). In an ideal situation without interocular suppression, participants’ perception would be similar to the composite image of stimulus items in both-eye arrays (i.e. the original array image). This perception is different from mixed perceptual rivalry dominance (cf.,^29^) in that all of the visual elements may be dominant simultaneously. We refer to this perceptual outcome as ‘composite binocular percept’ and it will be abbreviated as CBP. Then, we assessed the stability of CBP by measuring the average length of CBP incidences. Our predictions on the relative stability of each perception based on how it violates real-world constraints are shown in Fig. 1b.

## Results

### Experiment 1a: Binocular ensemble of coloured objects affect the incidence of binocular rivalry

**Figure 2:**
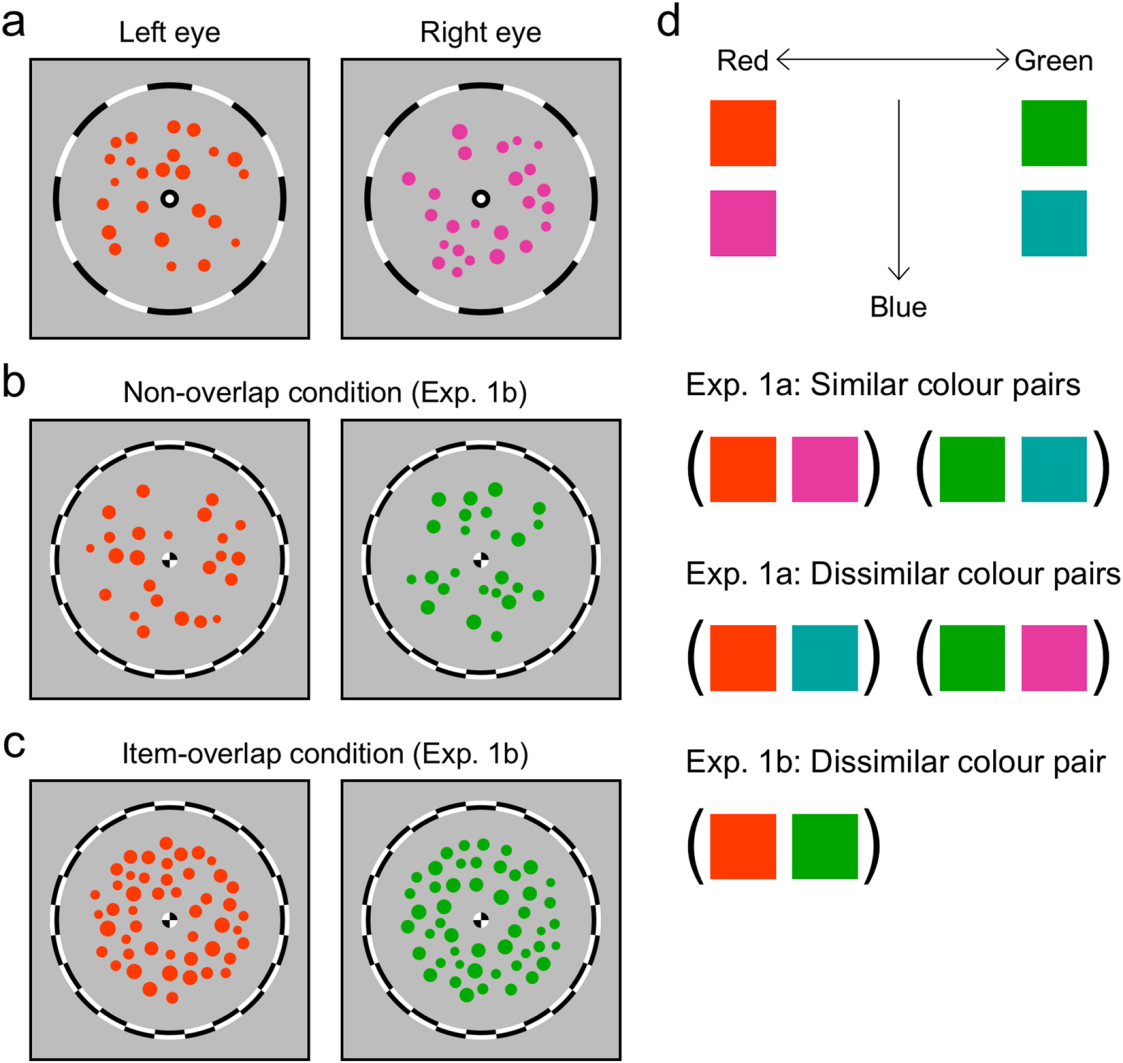
Example stimuli used in Experiment 1a are shown in (**a**), example stimuli of the non-overlap condition in Experiment 1b are shown in (**b**), and the item-overlap condition in (**c**). The colours of the circles in this figure are chosen to give a similar sense as the colours used in Experiments 1a and 1b. In the real Experiments, each participant adjusted colours so that they were subjectively equiluminant. We used two colours in the pure red-green scale and the other two colours made by adding a bit of blue to the pure red and pure green (**d**). Four pairs made from those four colours were tested in Experiment 1a. In Experiment 1b, only one pair of the pure red and pure green was tested.

**Figure 3:**
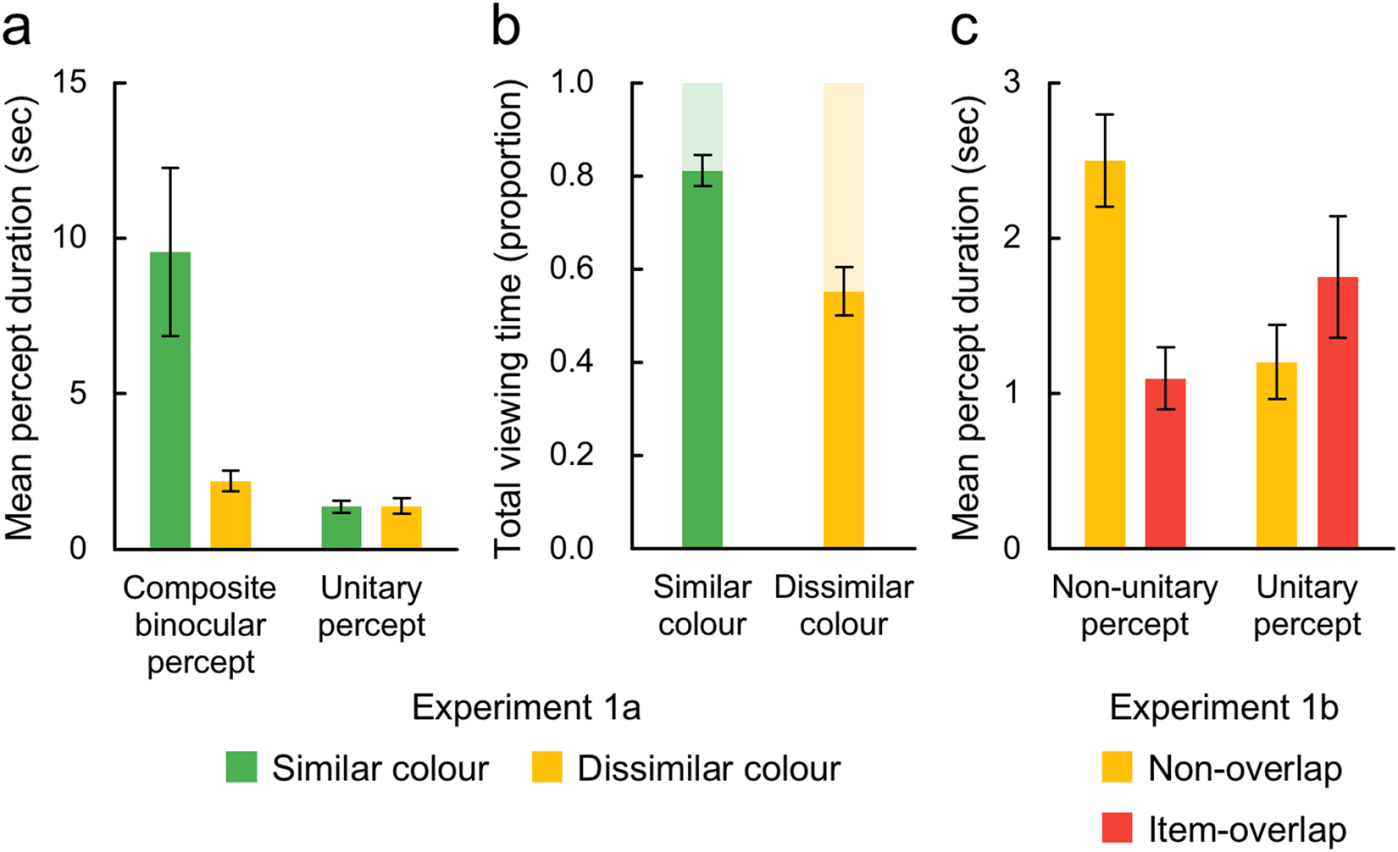
The mean duration of composite binocular percept (CBP) and unitary percept in Experiment 1a are plotted in (**a**). The proportions of the total viewing time of CBP are plotted in (**b**). The proportions of the total viewing time of unitary percept are complementary to those of CBP, and depicted as shaded bars over the solid bars. The mean duration of non-unitary and unitary percepts in Experiment 1b are plotted in (**c**). As the non-overlap condition in (**c**) and the dissimilar colour condition in (**a**) tested almost the same stimuli, their mean durations are similar. In all graphs, error bars indicate the standard error of mean.

Participants completed eight 90-second trials during which they viewed dichoptic arrays of coloured circles (Fig. 2a) and continuously reported colour(s) of the perceived stimuli by pressing and holding one or two of the two keys each assigned to one of the two colours of the dichoptic stimuli. In Experiment 1a, we tested the influence of colour similarity between the left- and right-eye arrays (Fig. 2d). As the CBP from arrays comprising similar colours would more likely arise within real-world viewing, we predicted that CBP would be more stable in the similar colour condition. As predicted, the mean duration of CBP was significantly greater under the similar colour condition (*F*(1, 9) = 8.277, *p* = 0.018, η_*p*_² = 0.479; Fig. 3a, composite binocular percept). During binocular rivalry, colour similarity did not influence the mean percept duration (*F*(1, 9) = 0.008, *p* = 0.931, η_*p*_² = 0.001; Fig. 3a, unitary percept). Furthermore, participants experienced CBP frequently in the similar colour condition (proportion of total viewing time: M = 0.81, SD = 0.03), and the proportion of time spent in the CBP state was higher in the similar colour condition relative to the dissimilar colour condition (*F*(1, 9) = 30.862, *p* < 0.001, η_*p*_² = 0.774; Fig. 3b). These differences are evident in the raw data of each participant (Figs S1 and S2). Unusual feature ensembles triggered binocular rivalry more frequently, presumably because they were indicative of unsuccessful binocular matches. Similarly, the high incidence of CBP would imply probable feature ensembles that promote binocular matching rather than interocular suppression. During CBP in the similar colour condition, participants’ perceptual experiences could be described as a coherent texture of circles having similar colours.

Experiment 1a focused on how the likelihood of feature combinations influenced the stability of CBP. Consequently, we discriminated CBP from mixed percept, which usually occurs during switches of perceptual dominance and is thus difficult to maintain stably over time. However, one might argue that mixed percepts could be stable without the apparent sense of perceptual changes. Recently, Klink and colleagues^30^ suggested that observers did not experience perceptual changes when they viewed a female face in one eye and a male face in the other eye. Rather, observers saw an androgynous face which would be a composite of the facial features in both-eye images. We thought that an androgynous face might be the composite of the parts of dichoptic face images, but which facial features from which-eye image contribute to the composite might change over time. There is a possibility that participants did not notice perceptual changes because the changes were happening outside the focus of attention. Brascamp and colleagues^31^ made this point by deploying occasional probe displays during dichoptic viewing of different random dot kinematograms (RDKs). In their study, observers did not notice perceptual changes to dichoptic same-colour RDKs, but when probed with coherent motion dots flashed during binocular viewing, they sometimes failed to see RDKs that were easily detected during perceptual dominance. These results suggest that dots viewed by one the eyes were suppressed interocularly. Accordingly, we felt compelled to compare CBP with mixed percept in our paradigm.

### Experiment 1b: Incompatible objects at the corresponding retinal locations produce an unusual binocular ensemble

In Experiment 1b, we compared CBP with mixed percept with a prediction that perception of mixed dominance would be less stable because it usually occurs in the process of perceptual switches. Participants performed the same tracking task as in Experiment 1a, this time with ensembles for which the spatial proximity of dichoptic stimulus items was manipulated. Stimulus items (i.e. coloured circles) were either prohibited from falling on corresponding retinal locations (Fig. 2b, non-overlap) or allowed to fall on corresponding retinal locations (Fig. 2c, item-overlap). When item-overlap was allowed, dichoptic stimulus items on corresponding retinal locations were incompatible, which would induce perception of mixed dominance rather than CBP. We predicted that mixed dominance (i.e. non-unitary perception in the item-overlap condition) would be less stable than CBP (i.e. non-unitary perception in the non-overlap condition). The colours of the circles were always highly dissimilar between the left- and right-eye arrays (Fig. 2d). As predicted, the mean durations of non-unitary percepts were brief in the item-overlap condition (*F*(1, 9) = 43.432, *p* < 0.001, η_*p*_² = 0.828; Fig. 3c, mixed perception). In addition, this item-overlap yielded longer durations of unitary colour perception that signify binocular rivalry (*F*(1, 9) = 9.116, *p* = 0.014, η_*p*_² = 0.503; Fig. 3c, unitary percept). In our framework, perceived ensembles during unitary perception are supported by a denser array in the item-overlap condition than in the non-overlap condition. This difference may have made unitary perception longer in the item-overlap condition. A denser array of circles experienced during unitary perception in the item-overlap condition would be very similar to the stable CBP in the similar colour condition of Experiment 1a.

### Experiment 1c: Similarity among coloured object ensembles promotes incidences of composite binocular percept

**Figure 4:**
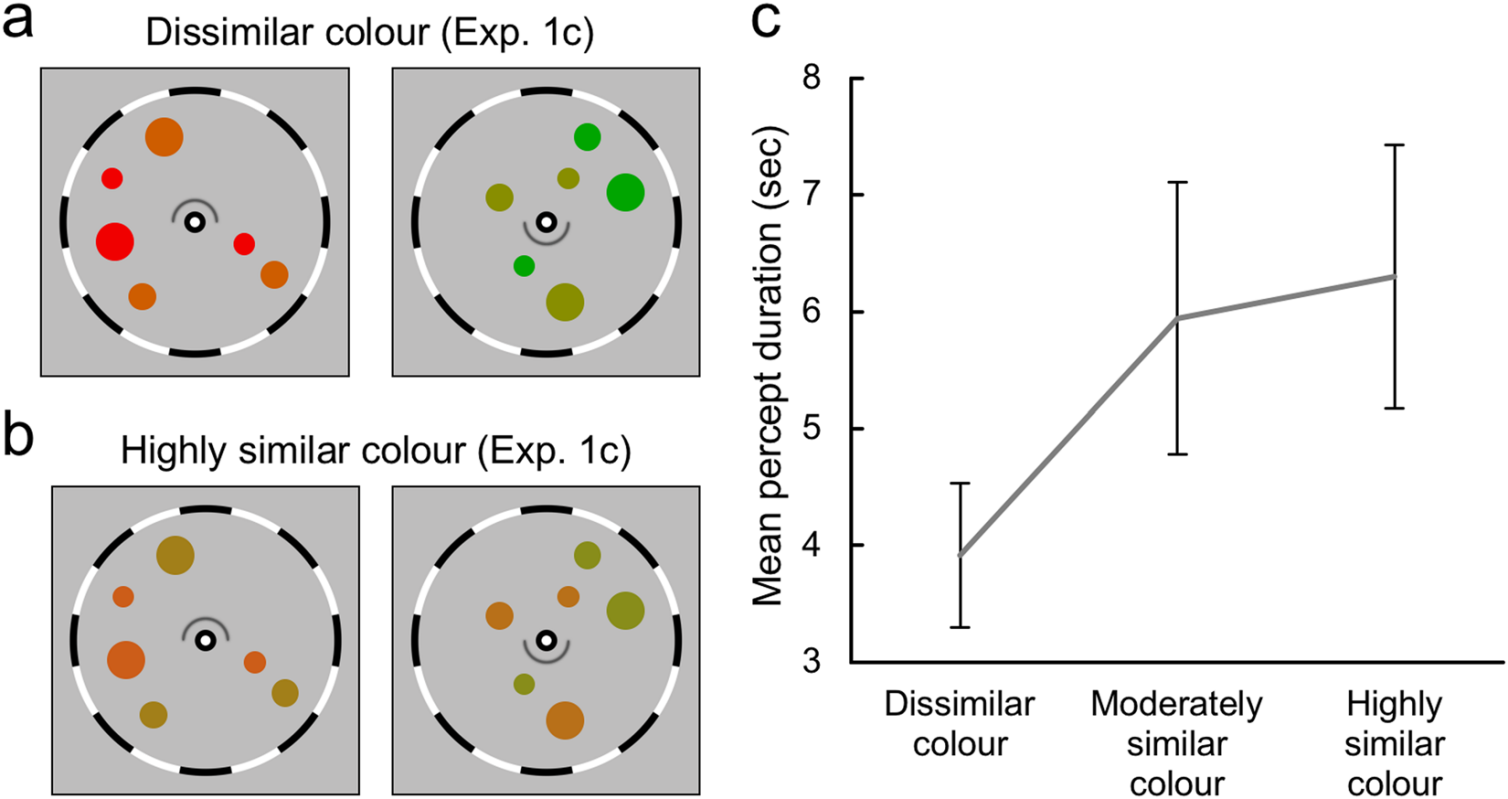
Conceptual example of dichoptic stimuli having dissimilar colour is shown in (**a**) and highly similar colour in (**b**). The mean durations of composite binocular percepts in Experiment 1c are plotted in (**c**). For the presentation purpose, coloured circles are drawn in relatively bigger sizes compared to the bulls-eye fixation points and fusion frames. In this experiment, circles in each eye could have one of the two colours yielding a total of four colours for the two eyes. Around the fixation point, upper and lower halves of a ring was presented in the separate eyes, and participants reported whether they saw an entire ring. Mean durations depending on the similarity between stimulus colour combinations are plotted in (**c**), and the raw data of participants’ responses are shown in Supplementary Figs S4 and S5. Error bars indicate the standard error of mean.

In Experiments 1a and 1b, circles viewed by one eye always had the same colour, and this arrangement allowed participants to report their perceptual state easily by monitoring colours of the visible circles. However, one concern of this approach may be that the stimuli in one eye do not have statistical variations in terms of colour. To address this concern, we ran Experiment 1c in which each circle viewed by each eye had one of two different colours, yielding a total of four colours for the two eyes. First, we generated eight subtle but distinguishable colours situated on the red-green axis, with the red/green luminance ratio varied to produce seven equal sized steps in that ratio ranging from red only (designated as colour 1) to green only (designated as colour 8), with these two end-point colours being subjectively matched in luminance. Then dichoptic colour pairs were created to produce rivalry between pairs with dissimilar colours (1/3 vs 6/8), pairs with moderately similar colours (2/4 vs 5/7) and pairs with highly similar colours (3/5 vs 4/6). These various colour combinations made it impractical to require participants to report perception based on colours, so we devised a different method that involved presenting the upper and lower halves of a ring to the separate eyes (Fig. 4a,b). Here the participant’s task was to press and hold a key when the entire ring was seen (corresponding to CBP) and to release the key when only the upper or lower part of the ring was visible. We again found evidence that the incidence of CBP increased with increasing colour similarity (*F*(2, 16) = 8.023, *p* = 0.004, η_*p*_² = 0.501; Fig. 4c). The raw data of participants’ responses are shown in supplementary figures (Figs S4 and S5). More importantly, mean percept durations of CBPs had linearly increasing trend with increasing colour similarity (*F*(1, 8) = 10.895, *p* = 0.011, η_*p*_² = 0.577).

The results of Experiments 1a through 1c suggest that the likelihood of binocular feature ensembles influences the likelihood of stable binocular combination. Our argument is, however, based on a conjecture that the eye-of-origin information is ignored in binocular feature ensembles, a reasonable thing to expect based on other evidence pointing to the reputed inability of people to identify which eye is being stimulated^32^. Still, if this were not the case for our displays, there remains a possibility that the *similarity* of monocular feature ensembles between dichoptic arrays caused this ensemble likelihood effect. In all three Experiments, the likelihood of a binocular feature ensemble was always correlated with the similarity between feature ensembles of dichoptic monocular arrays. When a feature ensemble of a superimposed image was probable (similar colour condition in Exp. 1a, highly similar colour condition in Exp. 1c), feature ensembles of dichoptic monocular arrays were similar. When a feature ensemble of a superimposed image was unusual (dissimilar colour condition in Exps. 1a and 1c, both item-overlap and non-overlap conditions in Exp. 1b), feature ensembles of dichoptic monocular arrays were dissimilar. Since the similarity between monocular ensembles was coupled with the likelihood of binocular ensemble in Experiments 1a through 1c, we felt it imperative to learn whether *unusual* binocular ensembles would trigger binocular rivalry when feature ensembles were matched between dichoptic stimulus arrays.

### Experiment 2: Unusual binocular ensembles trigger rivalry whether or not monocular ensembles are matched between dichoptic arrays

**Figure 5:**
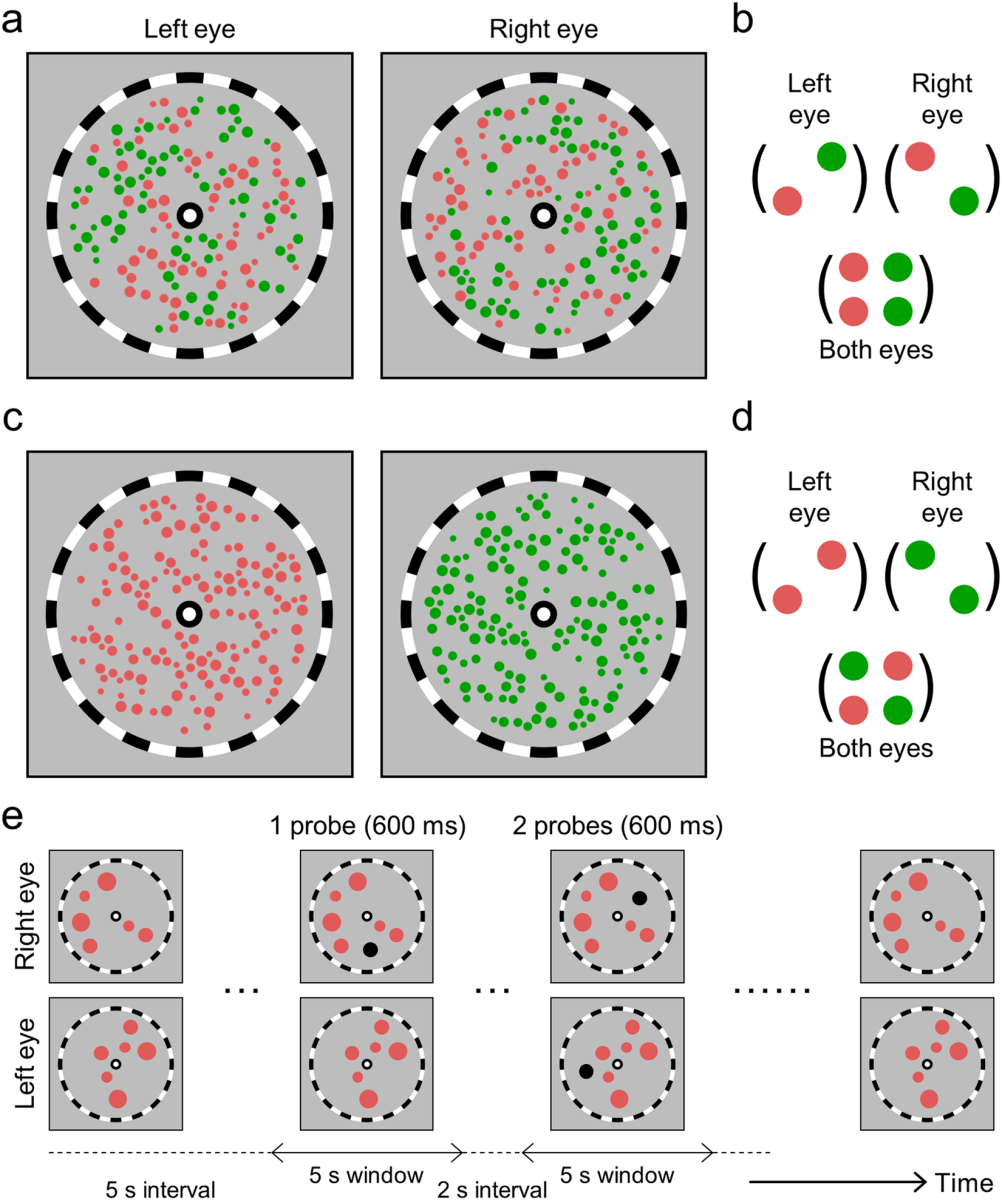
Example stimuli of the combined colour condition in Experiment 2 are shown in (**a**) and the dissimilar colour condition in (**c**). Stimuli in those two conditions are dissimilar in terms of dichoptic monocular ensembles yet similar in joint, binocular ensembles (**b**,**d**). The same colour condition is not depicted in this figure, but was tested in Experiment 2. The procedure of a single trial in Experiment 2 is shown in (**e**). Note that when two probes appeared simultaneously, they always appeared at different retinal locations in different eyes.

To match feature ensembles between dichoptic monocular arrays, we created dichoptic stimuli that had both red and green circles evenly distributed between the left- and right-eye arrays (Fig. 5a). In this combined colour condition, monocular feature ensembles would be similar in both-eye arrays (Fig. 5b) yet a binocular feature ensemble would be unusual as in the dissimilar colour condition (Fig. 5c,d). We wanted to learn whether the combined colour condition would trigger binocular rivalry, but the mixture of colours in this condition made it impossible for participants to indicate rivalry state based on the colour(s) of the perceived circles: both CBP and unitary percept would comprise red and green circles. To overcome this handicap, we turned to an indirect, probe technique that has been used before in studies of binocular rivalry (e.g.,^33^). We asked participants to detect the presentation of probe circle(s) and to report the number of simultaneous probes.

Participants completed twelve 90-second trials each containing six one-probe and six two-probe events (Fig. 4e). Probes were of sufficient contrast to be detected when presented during dominance, but sufficiently weak to preclude detection during suppression^34^. Interocular suppression would decrease participants’ performance of detecting one-probe events because a probe had 50% chance of appearing in the suppressed eye. In the case of two-probe events, in which each probe always appeared in different eyes, interocular suppression would decrease participants’ performance of discriminating the correct number of probes. One of those two probes was highly likely to appear within a region of suppression in one eye or the other. Thus, we used the conditional probability of the correct discrimination given that participants detected at least one of the probes (hereafter, discrimination rate) as a proxy index of interocular suppression. In addition, participants would have more chance of detecting at least one probe (hereafter, detection rate) when two probes appeared as one of those two probes was highly likely to appear within a region of dominance.

There were three levels of dichoptic stimulus condition (combined colour, same colour, and dissimilar colour), and our interest was to determine whether the combined colour condition would trigger binocular rivalry. For purposes of comparison, we administered the same probe technique during the same colour and dissimilar colour conditions. Still, a participant’s task was to report whether one or two probes appeared.

**Figure 6:**
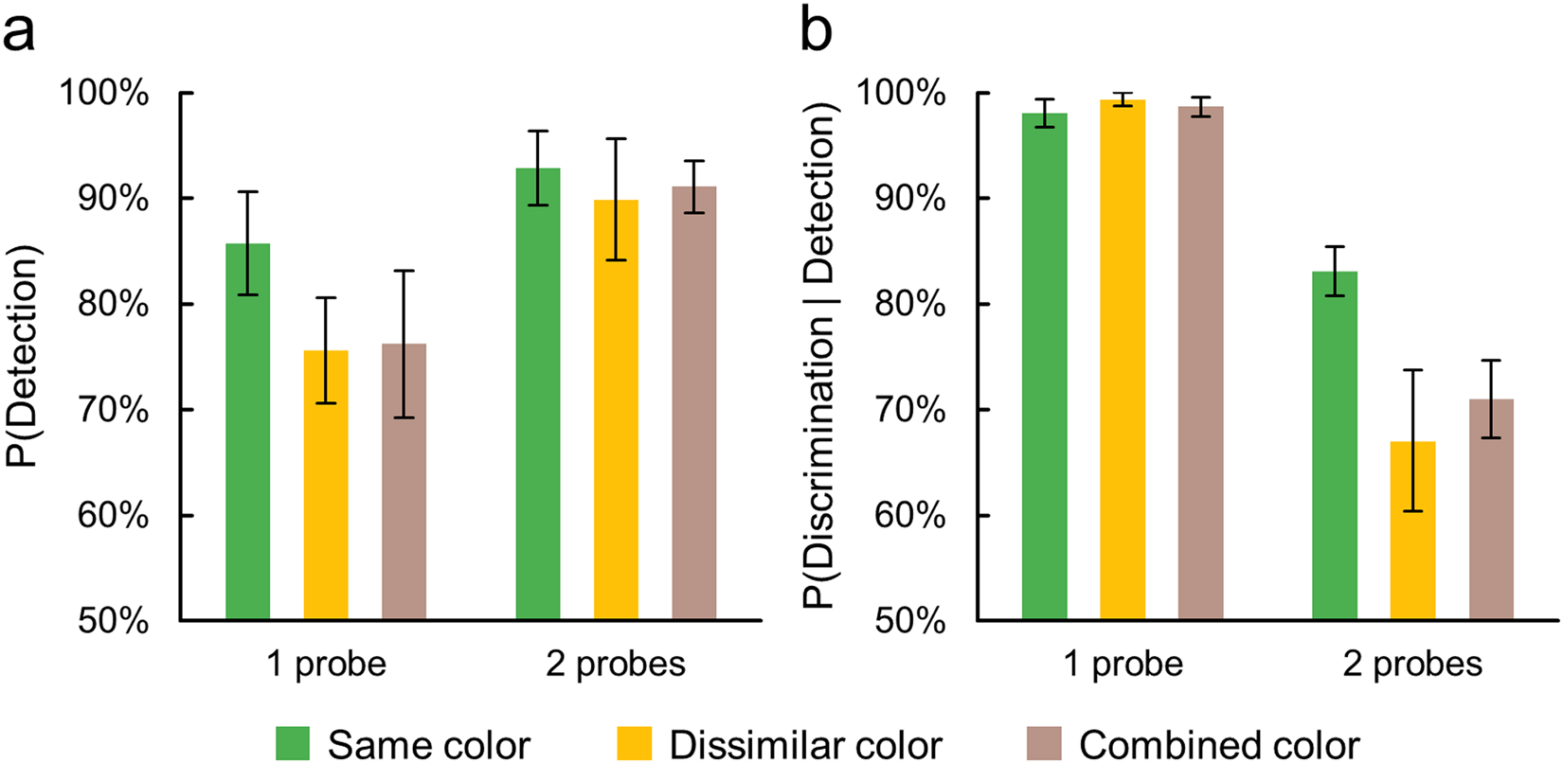
Detection rates of the one-probe and two-probe events in Experiment 2 are shown in (**a**), and discrimination rates in (**b**). Detection rates refer to the chance of any responses following the appearance of probes regardless of the number of probes. Discrimination rates refer to the conditional probability of reporting the correct number of probes given that participants detected at least one of the probes. In all graphs, error bars indicate the standard error of mean.

Figure 6 shows the results of Experiment 2. First, participants were more successful at detecting one-probe events during the same colour condition compared to detection performance during the dissimilar colour and the combined colour conditions (*F*(2, 12) = 5.200, *p* = 0.024, η_*p*_² = 0.464; Fig. 6a, 1 probe). Second, participants were more likely to miss one of the two probes in the dissimilar colour and combined colour conditions than in the same colour condition (*F*(2, 12) = 7.539, *p* = 0.008, η_*p*_² = 0.557; Fig. 6b). Finally, detection rates were higher when the two probes appeared simultaneously than when only one probe appeared (*F*(1, 6) = 26.266, *p* = 0.002, η_*p*_² = 0.814; Fig. 6a). This pattern of results makes sense if the dissimilar colour and the combined colour conditions engender binocular rivalry and, therefore, introduce the likelihood of one probe being rendered invisible by its being imaged within a region of suppression.

The results of Experiment 2 replicated the ensemble likelihood effect found in Experiment 1a. Detection performance on one-probe events and discrimination performance on two-probe events imply that participants experienced binocular rivalry more frequently in the dissimilar colour condition than in the same colour condition. Furthermore, the results of Experiment 2 suggest that binocular feature ensembles trigger binocular rivalry regardless of whether monocular ensembles were matched between the left- and right-eye arrays: participants experienced binocular rivalry in the combined colour condition as well as in the dissimilar colour condition. From these findings, we surmise that the brain may utilize feature regularities associated with binocular ensembles to determine whether the current binocular matches are probable. To offer a speculation, these results could imply that even a monocularly viewed image can induce rivalry on its own given the right conditions. Consistent with this implication, monocular stimuli indeed can induce a rivalry-like experiences^35^, the most well-known being monocular rivalry^36^: when two orthogonally oriented sinusoidal gratings, one red and the other green, are superimposed at the same location and viewed by both eyes, perceptual vividness of the two gratings fluctuates over time, following a temporal pattern resembling that measured during binocular rivalry^37^.

## Discussion

In this study, we explored a new possibility concerning the stimulus conditions that influence the binocular matching problem. In addition to the constraints already identified in earlier work^4–14^, we suggest that the visual system also evaluates the likelihood of valid, ecologically meaningful binocular integration based on the combined binocular ensemble of feature elements. Our results reveal that the combination of dissimilar ensembles intermingled within neighbouring vicinities constitutes an a priori unlikely spatial layout that triggers binocular rivalry (Experiments 1a through 1c: between corresponding areas of the two eyes, Experiment 2: within an area of one eye). Is this putative likelihood index of binocular image combinations feasible and useful based on known results?

Turning first to the question of feasibility, how might the visual system register the existence of unlikely, anomalous feature combinations in the two eyes’ views? Do there exist neural mechanisms capable of signalling feature combinations while preserving binocular difference information? Consistent with this idea is the notion of encoding binocular information by jointly implementing summation and differencing operations^38,39^, a conjecture that has received support from a recent psychophysical study by May and Zhaoping^40^ utilizing opposite-polarity images presented dichoptically. A differencing operator would respond strongly to that interocular image combination, thereby adapting over time. Immediately following adaptation, observers viewed dichoptic probe images that were carefully manipulated such that the summation and difference of the images resulted in gratings perceived to differ conspicuously in orientation. Indeed, observers reliably perceived the summation image after adapting to the opposite-polarity images, suggesting a unified channel that processes binocular differences. It is easy to imagine the balance of activity among the summation and differencing operations as embodying a likelihood estimate of binocular ensemble similarity.

In the context of our study and stimulus conditions, a feature ensemble can be construed as the image concomitant of a textured surface. This leads to the following question: How might the visual system synthesize a stable, coherent percept of a textured surface from dichoptic arrays of interocularly unpaired objects? Hints of an answer to this question can be found in a neural model of stereopsis proposed by Hayashi and colleagues^41^. Their model was designed to explain how the visual system assigns depth values to partially occluded visual objects. For instance, an object occluded by a plate held in front of the left eye may be visible from the viewpoint of the right eye only, yet we still gain a sense of three-dimensional depth associated with this unpaired monocular view of the object. The model developed by Hayashi and colleagues^41^ introduced interocularly unpaired point detectors and incorporated them with interocularly paired point detectors. As a result, unpaired visual elements could contribute to an object representation along with paired visual elements and could rival against each other. Dichoptic stimuli in the current study were made of numerous unpaired visual elements. In the context of ensembles as texture representations, those unpaired elements could form a coherent percept if their features were highly similar in the neighbouring detectors. On the other hand, if features were dissimilar, unpaired visual elements would produce binocular rivalry. The visual system could be sensitive to the likelihood that the joint ensemble assembled from stimuli in both eyes corresponds to a valid representation of what both eyes are viewing. When that likelihood is low, stable vision gives way to interocular suppression and rivalry.

As for usefulness, registering the likelihood of successful binocular matches could be particularly helpful when processing redundant visual information. Imagine, for instance, binocularly viewing an extended, textured surface such as a brick wall – the visual features defining that texture will be highly similar, which complicates the matching problem in the same way as does viewing a random-dot stereogram. Perhaps indexing binocular ensemble within a local region of large surfaces streamlines the process of computing the likelihood of valid binocular matches, with the outcome generalizing to neighbouring but unattended regions of space (in the same manner that disparity is assumed to vary gradually over space). We are intrigued by the possibility that this form of redundancy reduction might be a more efficient means for ignoring false matches, compared to active inhibition distributed over the entire representation of a surface. In this regard, we note that interocular suppression is, in fact, weak at locations where focused attention has been diverted^42–44^.

Finally, our supposition may provide a different way of conceptualizing previous results on interocular grouping^45,46^. In those studies, the visual system managed to derive specific interocular combinations encompassing parts of stimuli in both eyes that conformed to a spatially meaningful pattern. One way to explain interocular grouping is to assume that the visual system represents separate patterns in each eye’s views. The visual system may selectively stitch together neural representations of portions of the two patterns so as to promote overall global coherence (i.e. high likelihood). However, it would be inefficient to have separate pattern representations to deal with unlikely situations such as binocular rivalry. Another strategy would be to explore the domain of spontaneous combinations until one of those combinations achieves a high a priori likelihood. This solution seems inefficient, however, since blind trial-and-error testing of combinations could take a dangerously long time to stabilize on a solution. Another more robust means is to assume unified representation of unlikely feature combinations among neighbouring vicinities. The visual system may monitor certain parts from the opposite eye where perceived feature combinations are highly unlikely to happen. It is known that lateral interactions among neurons with neighbouring receptive fields could influence dominance status during binocular rivalry^47^. And during binocular rivalry, perceptual transitions originate at locations associated with pronounced interocular dissimilarity^48^. Along with the previous evidence, the current study suggests that plausible feature combinations could stabilize perceived images possibly though lateral interactions among neurons with neighbouring receptive fields. In this way, the visual system can steer the search process to portions of the neural image where likelihood is currently lowest.

In conclusion, we found that unlikely ensembles representing highly dissimilar features within immediately neighbouring areas can trigger binocular rivalry, suggesting that the visual system utilizes unlikely, anomalous binocular ensembles as evidence of unsuccessful matches of dichoptic images. Ensemble likelihood has its own role in achieving binocular stable visual perception: it promotes the visual system to establish binocular correspondence and produce three-dimensional impressions using disparity information. In natural viewing, probable ensembles are likely to be the products of good stimulus correspondence. If the same objects are imaged in the two monocular views, binocular ensembles from joint features would be highly probable. In this situation, high ensemble likelihood may serve as an index of likely binocular matching success.

## Methods

### Participants

Ten naïve, volunteer participants along with two of the authors (OC & RB) participated in Experiment 1a. Five of them participated from Vanderbilt University and the other seven from Yonsei University. Two participants in Yonsei University who did not experience percept changes in more than one trial were excluded from analyses because it would be impossible to quantify their percept durations in those trials. Their responses are depicted in the supplementary figure (Fig. S3). Ten participants excluding those two also volunteered for Experiment 1b. As the effects of our manipulations (colour similarity in Exp. 1a, item-overlap vs. non-overlap in Exp. 1b) are literally observable in the raw data (Figs S1–S7), we considered a total of ten participants to ensure a sufficient number of participants. Ten participants including one of the authors (OC) volunteered for Experiment 1c in Yonsei University. Seven participants including two of the authors (OC & SCC) volunteered for Experiment 2 in Yonsei University. In all four Experiments, all participants had normal or corrected-to-normal vision, and gave written informed consent. All procedures were approved by the Vanderbilt University Human Research Protection Program or Institutional Review Board of Yonsei University, and the study was carried out in accordance with the Code of Ethics of the World Medical Association (Declaration of Helsinki).

### Apparatus

Stimuli were generated using MATLAB (MathWorks, Natick, MA) with the Psychtoolbox-3 extension^49,50^, and non-overlapping placement of the stimuli was determined using custom-made functions (MATLAB code available at http://github.com/oakyoon/pretina-fabric).

At Vanderbilt University, stimuli were presented on a single, gamma-corrected CRT monitor (Sony CPD-E540, refresh rate: 100 Hz) in a dark room. Participants viewed the monitor through a four-mirror stereoscope that presented the left and right halves of the monitor to participants’ left and right eyes, respectively. Alignment of stimuli is described in a following section. Participants’ head position was secured using a head-and-chin rest. Viewing distance defined as the length of the optical path from eye to screen was 83.82 cm.

At Yonsei University, stimuli were presented on two gamma-corrected CRT monitors (Sun Microsystems GDM-5410, refresh rate: 85 Hz) in a dark room. The luminance of the two monitors was equalized using custom-made gamma correction functions (MATLAB code available at http://github.com/oakyoon/vcc-gamma). A two-mirror stereoscope reflected images of each of the two monitors to the corresponding retinal location of each eye. Participants’ head position was secured using a head-and-chin rest. In Experiments 1a through 1c, the two mirrors were fixed and the viewing distance through the mirrors was 65 cm. In Experiment 2, participants were to push and pull a metal plate with the two mirrors fixed on it. This push/pull adjusted the horizontal location of the reflected images of the two monitors and the participants adjusted the metal plate until the images of the two monitors fell on the corresponding retinal locations. Because of this adjustment, the length of the optical path differed for each participant, and it was 60 cm on average.

### Stimuli and Design

#### Experiment 1a

The binocular stimuli comprised 24 circles (Fig. 2a) in each eye. The size of each circle was sampled from a rectangular distribution based on psychological size^19,51^. The diameter of the average-sized circle was 0.2° and the standard deviation of diameters was 0.04°. The circles were randomly placed inside an annular window (outer diameter: 2.8°, inner diameter: 0.6°) with the constraint that the spacing among the circles (both within and between the eyes) should be at least 1 pixel. Thus, the circles did not contact any other circles in both eyes. To promote binocular fusion, identical fusion frames and fixation points were presented to both eyes.

The circles in one eye had the same hue and that hue could be one of the four colours (red, green, bluish red, and bluish green). Red (45.80 cd/m² at Vanderbilt, 21.78 cd/m² at Yonsei) was designated as the reference colour and each participant matched the subjective brightness of the other colours to the red colour using a flicker minimization method^52^. The average luminance of the three colours was as follows: green (53.66 cd/m² at Vanderbilt, 23.73 cd/m² at Yonsei), bluish red (48.02 cd/m² at Vanderbilt, 22.31 cd/m² at Yonsei), and bluish green (60.73 cd/m² at Vanderbilt, 25.98 cd/m² at Yonsei). The two bluish colours contained a predefined amount of blue (9.60 cd/m² at Vanderbilt, 3.22 cd/m² at Yonsei). The circles were not equiluminant with respect to the background: they all had positive luminance contrast to the grey background (31.20 cd/m² at Vanderbilt, 14.98 cd/m² at Yonsei). The circles were equiluminant with respect to one another to the extent that the flicker minimization procedure was successful.

Experiment 1a tested two levels of colour similarity, similar colour and dissimilar colour. Two similar and two dissimilar colour pairs were made from the four colours (Fig. 2d). Red and bluish red, green and bluish green pairs were used in the similar colour condition. Red and bluish green, green and bluish red pairs were used in the dissimilar colour condition. Each pair was tested twice and the colours were switched between the eyes across repetitions. Participants completed eight trials, resulting in four trials per each condition.

#### Experiment 1b

The binocular stimuli comprised either 24 circles in the non-overlap condition (Fig. 2b) and 48 circles in the item-overlap condition (Fig. 2c) in each eye. The size of each circle was sampled in the same way as in Experiment 1a. In the non-overlap condition, the circles were placed in the same way as in Experiment 1a. In the item-overlap condition, the circles were randomly placed with the constraint that circles were prohibited from contacting other circles within the same eye’s view. Thus, circles could partially overlap between the eyes (overlap was ~30% of the total area of the circles on average).

The circles viewed by one eye had red colour and circles viewed by the other eye had green colour (Fig. 2d). Each participant matched the subjective brightness of the green to the reference red (45.80 cd/m² at Vanderbilt, 21.78 cd/m² at Yonsei). The average luminance of the green was 56.26 cd/m² at Vanderbilt and 25.15 cd/m² at Yonsei.

Experiment 1b tested item-overlap vs. non-overlap conditions. The colours of the circles were always dissimilar between dichoptic arrays. Participants completed eight sessions, resulting in four sessions per each condition.

#### Experiment 1c

Stimuli in Experiment 1c were the same as in Experiment 1a except for the colours of the circles and for the addition of the upper and the lower halves of a ring presented around the fixation point in the left eye and in the right eye, respectively (Fig. 4a,b). These dichoptically viewed upper and lower halves would be binocularly integrated as a closed ring (diameter: 0.7°) when participants were experiencing CBP. Experiment 1c tested three levels of colour similarity. Dissimilar, moderately similar, and highly similar colour combinations were made from eight subtle but distinguishable colours which were situated on the red-green axis (see Supplementary Figs S4 and S5 for example colours). Participants completed twelve sessions, resulting in four sessions per each colour similarity condition.

#### Experiment 2

The stimuli comprised 150 circles viewed by each eye (Fig. 5a,c). The angular subtense of a diameter of a circle was drawn from a set of five values (0.24°, 0.29°, 0.34°, 0.38°, and 0.42°) with the constraint that each diameter was assigned to exactly 30 circles. The circles were randomly placed in the same way as in Experiment 1a but the annulus window was larger (outer diameter: 9.2°, inner diameter: 1.4°). The fusion frame (inner diameter: 10.0°, thickness: 0.4°) and fixation point (diameter: 1.0°) were increased in size accordingly. The colours of the circles were predefined red (13.91 cd/m²) and green (13.92 cd/m²), and the background was uniform grey (7.00 cd/m²). The red and green colours were determined in a separate pilot experiment so that their luminance and subjective brightness were similar to each other. The probes were dark grey circles (diameter: 0.34°, 4.16 cd/m²), and probe luminance values were determined in a pilot experiment. The probes had negative luminance in contrast to the background, which was the opposite polarity to the dichoptic stimuli. Probes were easily detectable when presented in the region of dominance, but were insufficient in magnitude to disrupt interocular suppression.

Experimental conditions were defined by the binocular colour combination of circles. In the combined colour condition (Fig. 5a), half of the circles seen by each eye were red and the other half were green. The distribution of the sizes was the same between red and green circles: 15 red and 15 green circles viewed by one eye had the same size. In the dissimilar colour condition (Fig. 5c), one eye viewed red circles and the other eye viewed green circles. Note that the joint feature ensembles were similar between the combined colour and the dissimilar colour conditions (Fig. 5b,d). In the same colour condition, both eyes viewed circles of the same colour. Each condition was tested four times and the colours were switched between the eyes across repetitions. Thus, the participants completed twelve trials.

### Tasks and Analyses

#### Binocular alignment task (Experiments 1a, 1b, and 1c)

Before each experiment, custom-designed software implemented a refined version of the cover/uncover test to achieve stable binocular alignment of the left- and right-eye images. Using computer keys, participants moved the left- and right-eye fusion frames by small steps repeatedly until the frames in both eyes appeared centred and perfectly overlapped within the visual field. This adjustment procedure was repeated multiple times while one of the images remained visible continuously and the other flashed on/off briefly. Only after achieving relative adjusted positions that produced stable alignment were the associated x/y coordinate values saved and used for positioning those frames during an experiment.

#### Brightness matching task (Experiments 1a, 1b, and 1c)

At the start of Experiments 1a and 1b, participants performed the subjective brightness matching task. In Experiment 1a, the subjective brightness matching task required participants to perform four colour matches. First, participants matched green to the reference red. Two other colours, bluish red and bluish green, were initialized by adding a predefined amount of blue to the red and green. Then, participants adjusted the luminance of bluish red until it matched the brightness of pure green (i.e. flicker was minimized), and they adjusted the luminance of bluish green until it matched the brightness of pure red. We used blue to shift the two bluish colours from pure red and from pure green, while maintaining their distance in the red-green dimension (Fig. 2d). Finally, participants matched bluish red and bluish green. Since the bluish colours were already matched to pure red and pure green, this final stage required minimum adjustment. In Experiment 1b, participants adjusted the green’s luminance until it matched red and they did so on three successive times, with the average of the three matches then being used in the perceptual tracking task. At the end of the matching task, participants viewed the matched colours and verbally confirmed that the colours were distinguishable and similarly salient.

#### Perceptual tracking task (Experiments 1a and 1b)

Once the colour matching procedure was completed, participants performed eight trials each lasting 90 seconds. Before starting each trial, an instruction text assigned the two colours to be viewed on that trial to the left and right arrow keys, and participants were to take time to memorize colour-key mappings carefully. Participants started a trial by pressing the space bar. Then, binocular stimuli appeared and rotated slowly (0.67 rpm) around the fixation point to prevent adaptation. During the trial, participants continuously reported their percept using the left and right arrow keys. When experiencing circles of one colour exclusively, participants pressed and held the arrow key assigned to that colour. When experiencing circles of both colours, participants pressed and held both arrow keys. The direction of rotation was randomized across trials, and the order of colour pairs was randomized across participants.

We divided a 90-second trial into several phases depending on the reported percept. A phase was a duration during which participants maintained the same key press combination. Certain phases were excluded from analysis. Phases with no key press and phases shorter than 50 ms were excluded because those phases were likely to happen between key changes. Phases at the end of each session were excluded because not a percept change but the end of session terminated the last phase. Then, phases were categorized into unitary and non-unitary percepts (Exp. 1a: CBP, Exp. 1b: CBP in the non-overlap condition, mixed percept in the item-overlap condition). A unitary percept phase referred to the phases in which participants saw stimuli in one eye exclusively. A non-unitary percept phase referred to the phases in which participants saw a mixture of stimuli in both eyes. We mainly analysed the mean durations of non-unitary and unitary percepts in Experiments 1a and 1b. In addition, we analysed the proportion of total viewing time of the CBP in Experiment 1a. Note that the proportion of total viewing time between the CBP and unitary percept is complementary to each other.

#### Perceptual tracking task (Experiment 1c)

In Experiment 1c, participants performed twelve trials each lasting 90 seconds. Participants started a trial by pressing the space bar, then binocular stimuli appeared and rotated slowly (0.67 rpm). At the same time, the upper and lower halves of a ring were presented around the fixation point (Fig. 4a,b), and participants pressed and hold a key when the entire ring was seen (corresponding to CBP) and to release the key when only the upper or lower part of the ring was visible. The upper and lower halves of a ring did not rotate during a trial.

#### Probe discrimination task (Experiment 2)

In each trial, participants viewed the dichoptic stimuli (red and green circles) for 90 seconds, and at unpredictable times during the trial, probe(s) briefly appeared multiple times. Since we wanted to investigate the tendency to suppress stimuli in one eye rather than the stimuli themselves, we presented the probes in empty space among the circles. The probes were newly presented dark grey circles. Following their appearance, the probe(s) remained on the screen for 500 ms, with probe onsets and offsets being ramped up and down by a sigmoid function. There were twelve probe events in one trial. On half of the probe presentations, a single probe was presented to one eye (Fig. 5e, 1 probe), and on the other half of the probe presentations, two probes were presented simultaneously (one to each eye; Fig. 5e, 2 probes). Participants were instructed to report immediately whenever they detected presentation of one or two probes using the 1- and 2-keys on a numpad. During the 90-second trial, a probe event could occur within a five-second window and successive windows were separated by two-second intervals. The first window in each trial started five seconds after the beginning of the trial. With the exception of OC (an author), participants were unaware how many probes to expect nor when they might appear; they only knew that sometimes two probes would appear at different locations simultaneously while at other times only a single probe would appear.

We calculated detection and discrimination rates associated with one-probe and two-probe events. Detection refers to any response within two seconds following a probe event. Therefore, reporting one probe to two-probe events and reporting two probes to one-probe events were considered as a successful detection. Discrimination refers to the responses that reported the correct number of probes within two seconds. We calculated the conditional probability of correct discrimination given that participants detected at least one of the probes. We used this conditional probability as the discrimination rate, because this conditional probability provides an indirect confirmation that binocular rivalry was occurring. Under binocular rivalry, participants were more likely to fail to detect one of the two probes because it was more likely to appear within a region of suppression. We are cognizant of the fact that rivalry, when it was happening, might not encompass the entire region of one eye’s view – dominance and suppression could be happening within different regions of a given eye’s view. Still, we could compare probe detection and discrimination performance under the combined colour condition to that under the patent rivalry condition to infer whether rivalry was happening under the former.

### Data availability statement

The datasets of Experiments 1a and 1b generated or analysed during this study are included in this published article and its Supplementary Information file. The datasets of Experiment 2 generated during and/or analysed during the current study are available from the corresponding author on reasonable request.

## Electronic supplementary material

Supplementary Information
