# Supplementary material for: Composite binocular perception from dichoptic stimulus arrays with similar ensemble information
Source: Sci Rep. 2018 May 29;8:8263. doi: 10.1038/s41598-018-26679-9 (PMC5973937; doi:10.1038/s41598-018-26679-9)
Supplement: Supplementary file 1 — Supplementary Information [file 41598_2018_26679_MOESM1_ESM.docx]

Composite binocular perception from dichoptic stimulus arrays with similar ensemble information.

Oakyoon Cha1, Randolph Blake2, and Sang Chul Chong1,3,*

1Graduate Program in Cognitive Science, Yonsei University, Seoul, 03722, Korea

2Department of Psychology and Vanderbilt Vision Research Center, Vanderbilt University, Nashville, TN 37240, USA

3Department of Psychology, Yonsei University, Seoul, 03722, Korea

*scchong@yonsei.ac.kr


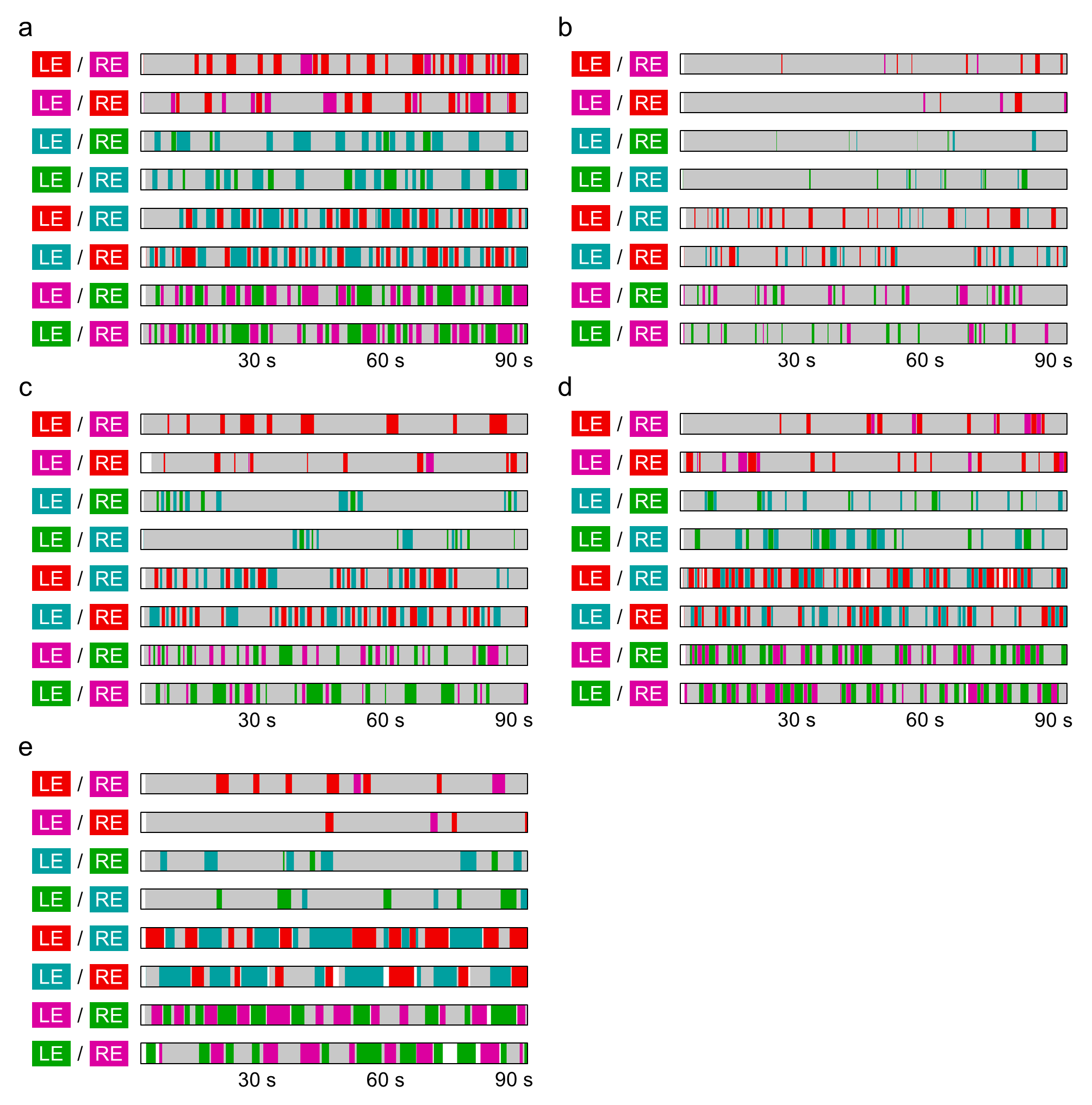


**Figure S1.** Raw data of five participants (a-e) in Experiment 1a. These five participants were tested in Vanderbilt University and included in the analyses. Each row shows participants’ key responses against 90-second duration. In the left side of each row, two boxes are filled with colours of the stimuli that was presented to the left eye (LE) and the right eye (RE). Coloured areas in each row are percept phases in which participants reported seeing that colour. Areas in grey colours are percept phases in which participants reported seeing both colours. Uncoloured, white areas indicate that participants did not press any keys during that period. In each panel, upper four rows show responses in the similar condition, and bottom four rows in the dissimilar colour condition. Two (d, e) of these five are from the authors OC and RB.


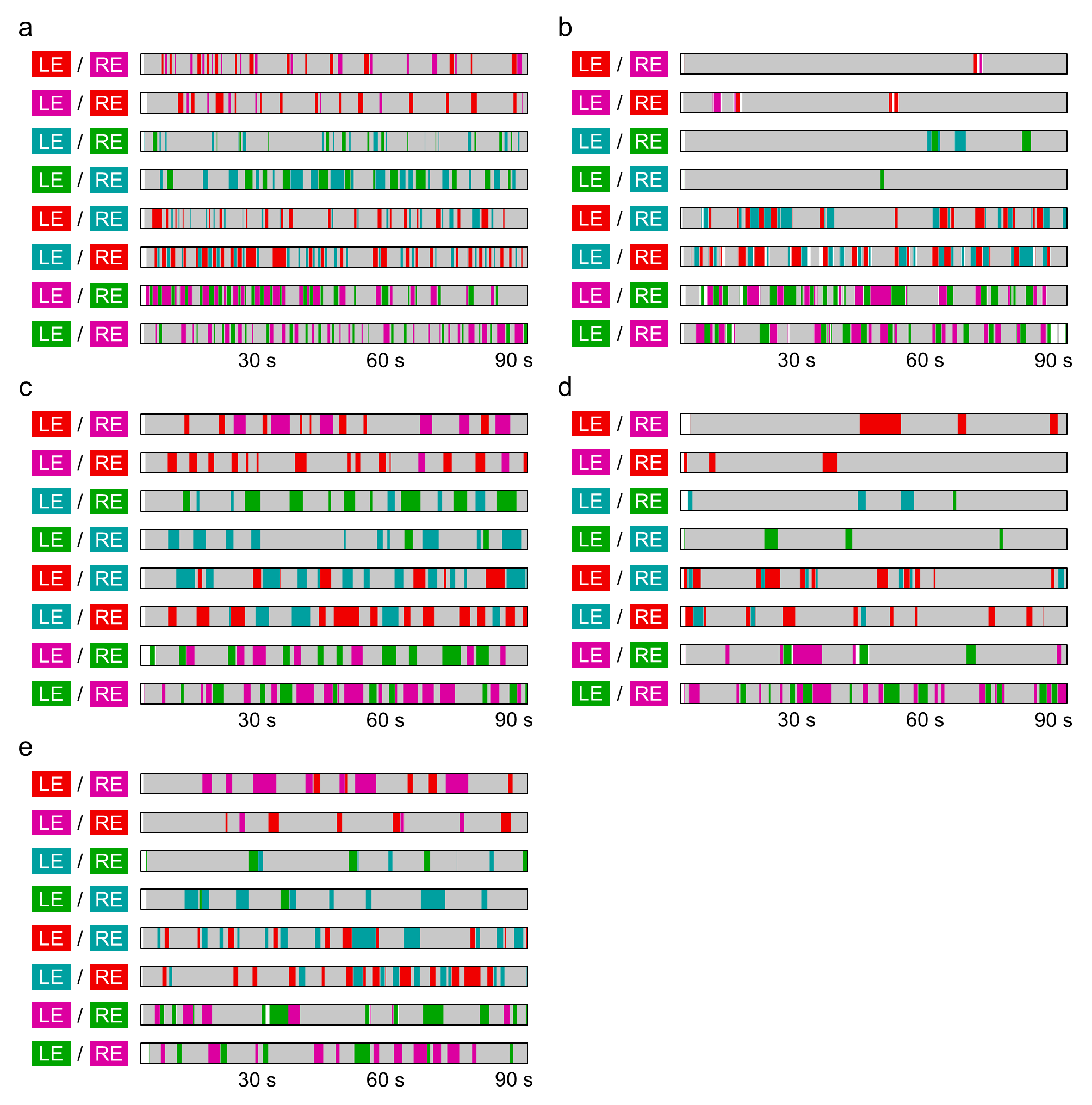


**Figure S2.** Raw data of five participants (a-e) in Experiment 1a. These five participants were tested in Yonsei University and included in the analyses. In each panel, upper four rows show responses in the similar condition, and bottom four rows in the dissimilar colour condition.


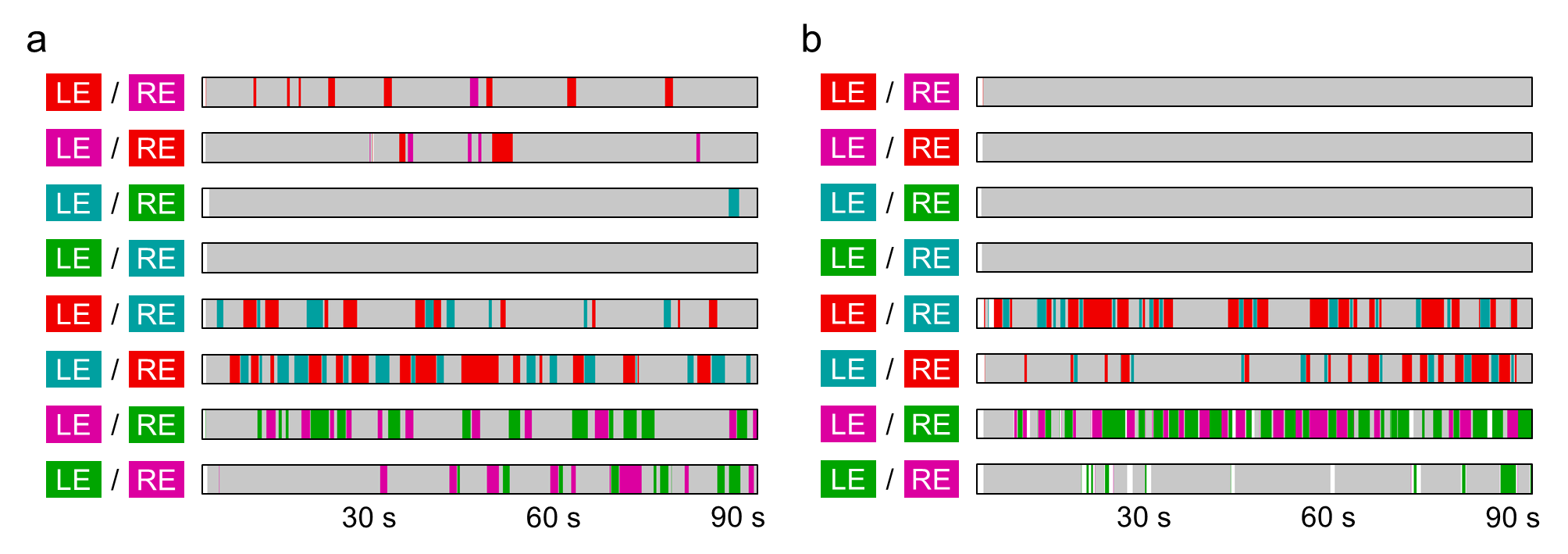


**Figure S3.** Raw data of two participants (a, b) in Experiment 1a. These two participants were tested in Yonsei University and excluded in the analyses due to trial-long mixed percepts. In one or more trials, they experienced mixed percept for whole 90 seconds. See third row of participant (a) and first, second, third, and fourth rows of participant (b). Still, these trial-long mixed percepts only happened in the similar colour condition (upper four rows).


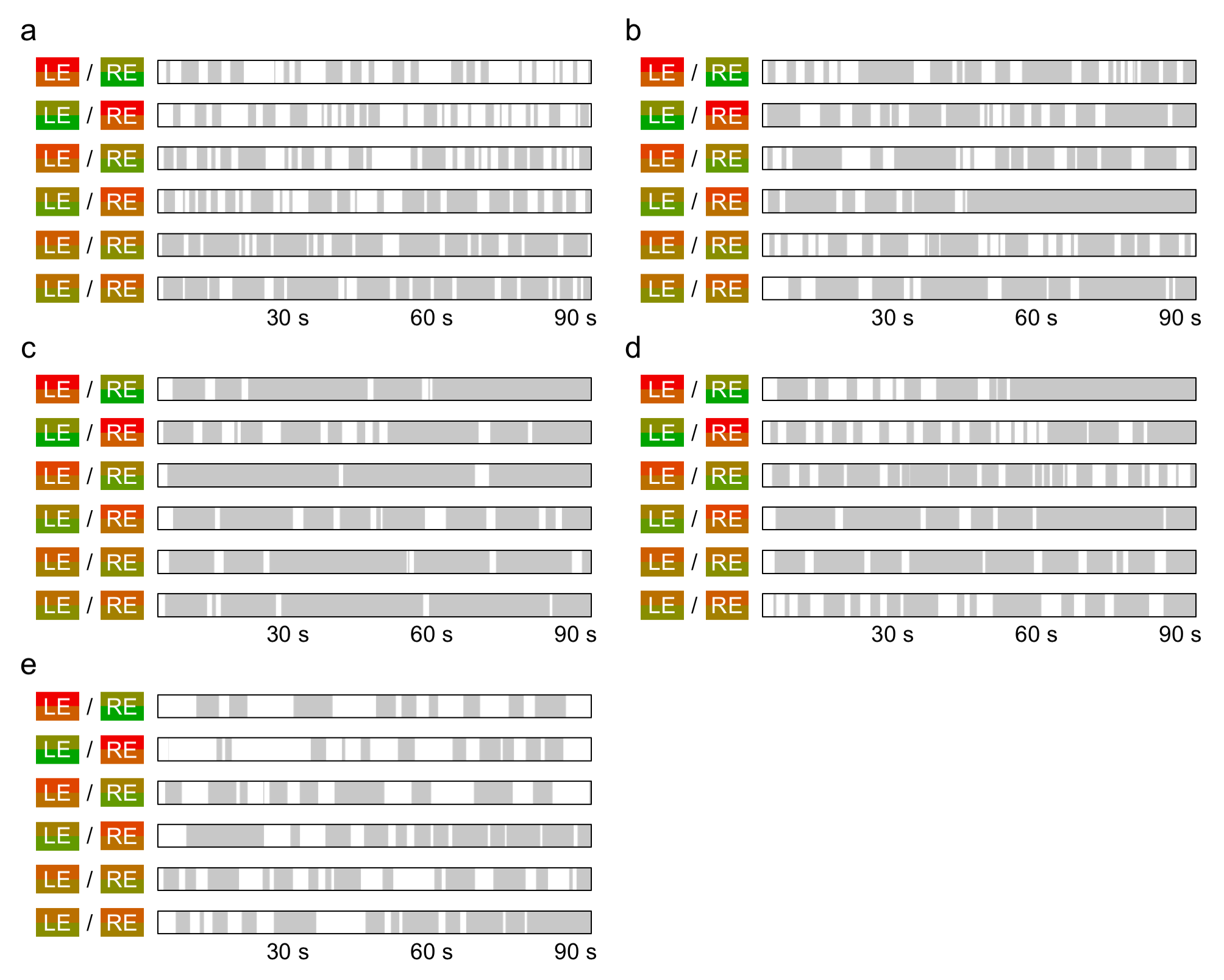


**Figure S4.** Raw data of five participants (a-e) including the author OC (a) in the control experiment. Each row shows participants’ key responses against 90-second duration. In the left side of each row, two boxes are filled with two colours of the stimuli that was presented to the left eye (LE) and the right eye (RE). In this experiment, participants only reported incidences of composite binocular percepts (CBPs) by monitoring a ring around the fixation point (see Fig. S4a). Incidences of CBPs are filled with grey colours. In each panel, the top two rows show participants’ responses to the binocular stimuli with dissimilar colours, the middle two rows show responses to the stimuli with moderately similar colours, and the bottom two rows show responses to the stimuli with highly similar colours.


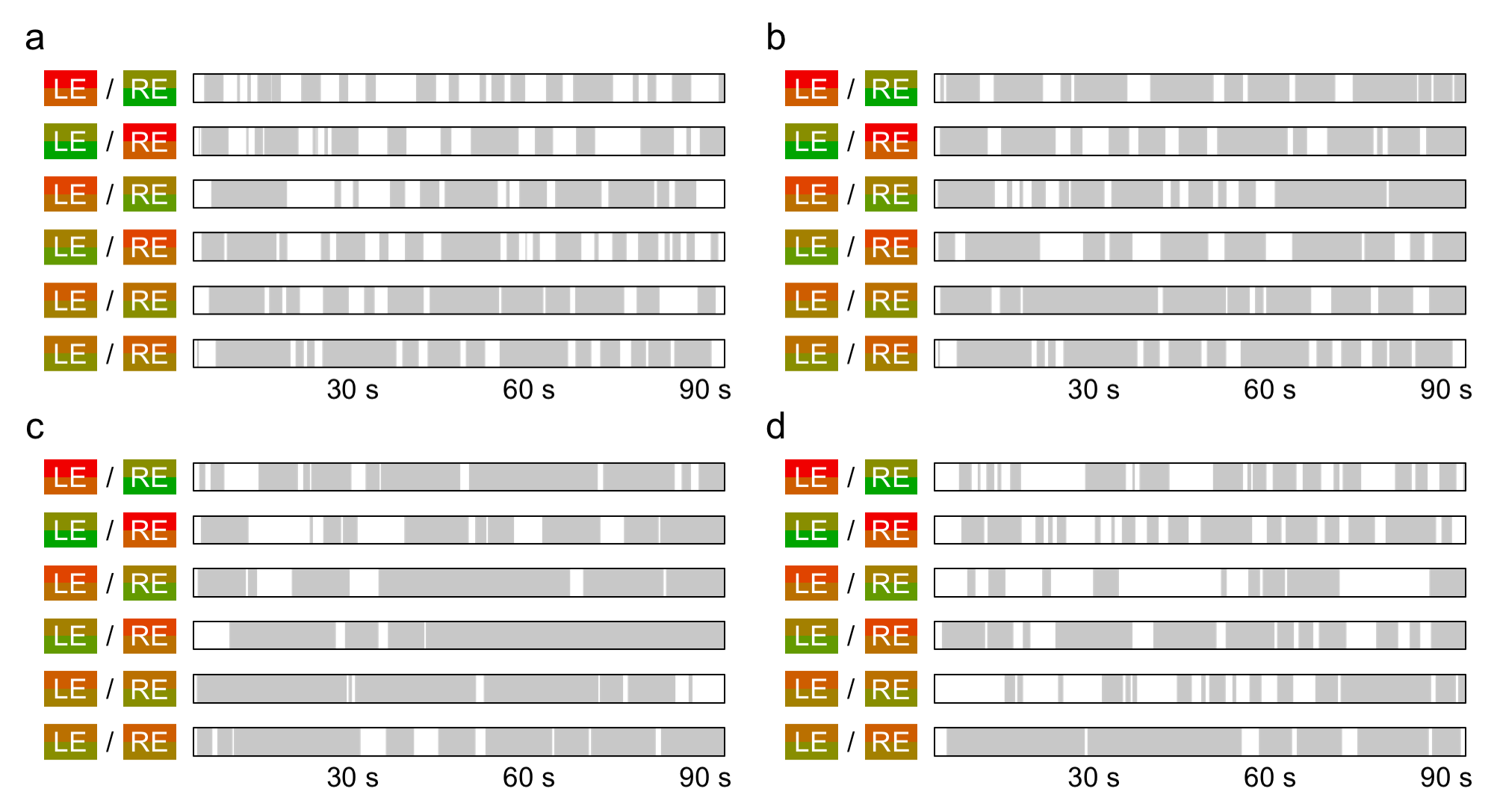


**Figure S5.** Raw data of four participants (a-d) in the control experiment. Incidences of composite binocular percepts are filled with grey colours. In each panel, the top two rows show participants’ responses to the binocular stimuli with dissimilar colours, the middle two rows show responses to the stimuli with moderately similar colours, and the bottom two rows show responses to the stimuli with highly similar colours.


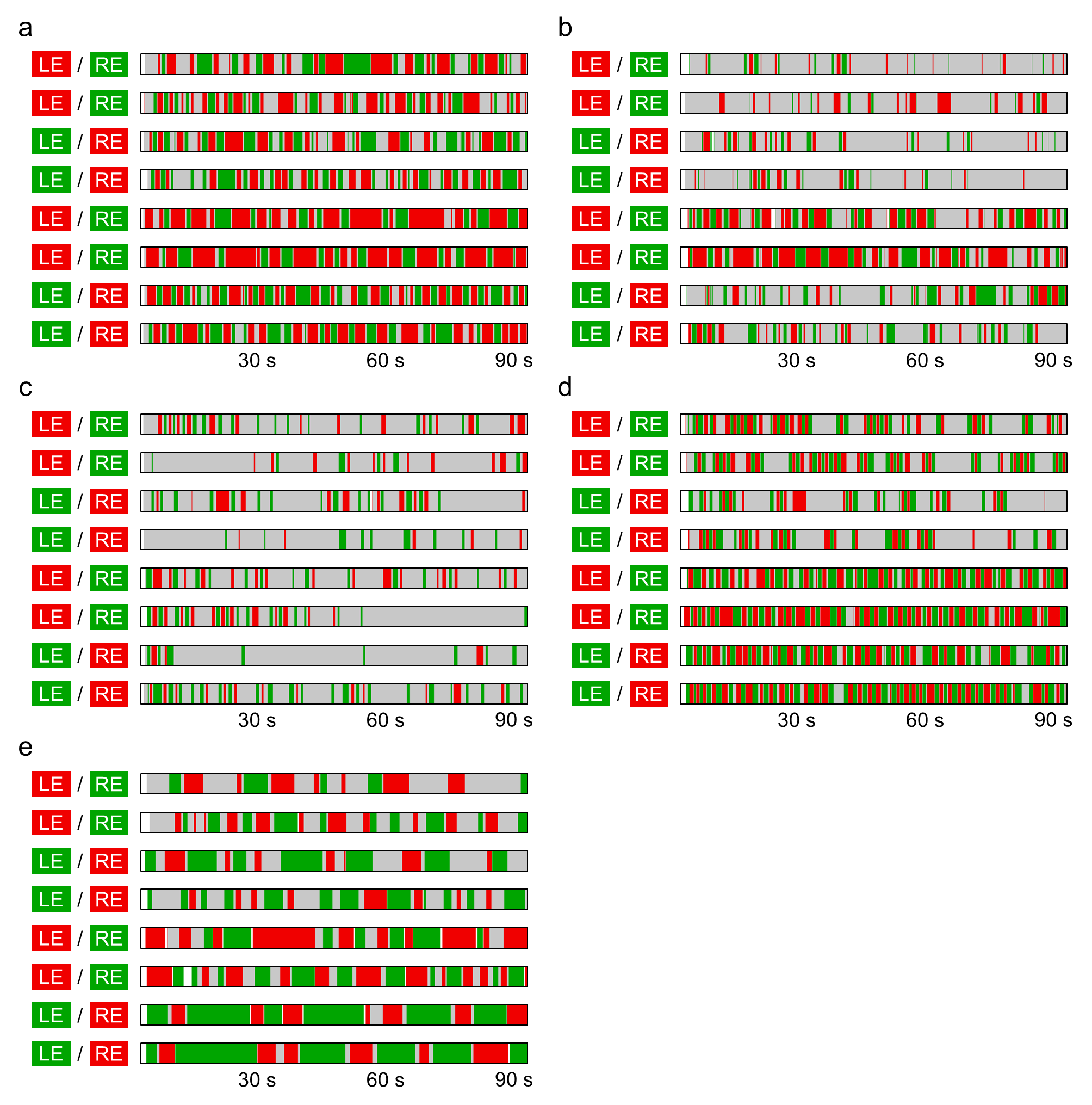


**Figure S6.** Raw data of five participants (a-e) in Experiment 1b. These five participants were tested in Vanderbilt University and included in the analyses. In each panel, upper four rows show responses in the non-overlap condition, and bottom four rows in the item overlap condition. Two (d, e) of these five are from the authors OC and RB.


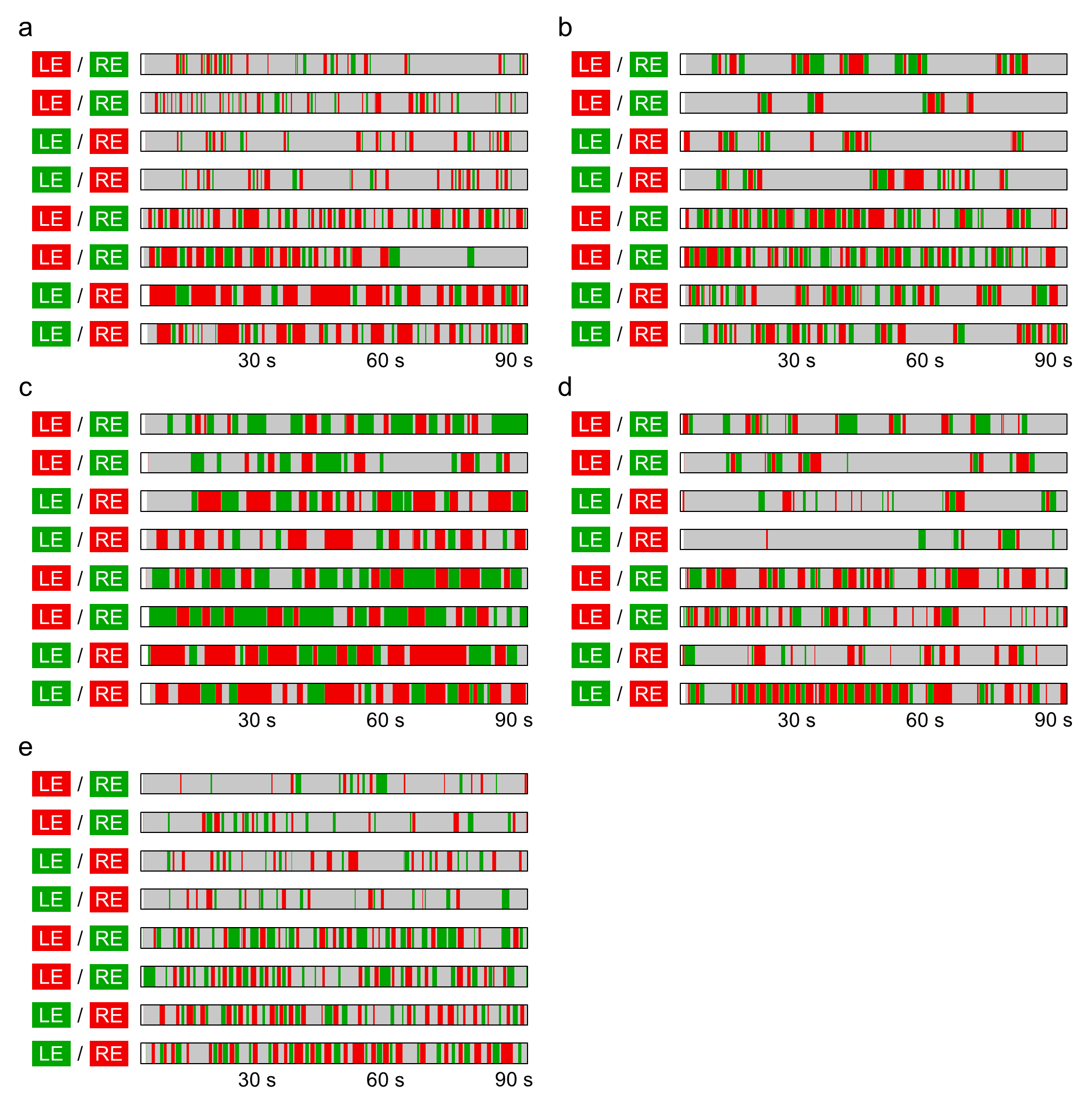


**Figure S7.** Raw data of five participants (a-e) in Experiment 1b. These five participants were tested in Yonsei University and included in the analyses. In each panel, upper four rows show responses in the non-overlap condition, and bottom four rows in the item overlap condition.
